# Supplementary material for: A System for Assessing Dual Action Modulators of Glycine Transporters and Glycine Receptors
Source: Biomolecules. 2020 Nov 30;10(12):1618. doi: 10.3390/biom10121618 (PMC7760315; doi:10.3390/biom10121618)
Supplement: Supplementary file 1 [file biomolecules-10-01618-s001.pdf]

Supplementary Figures and Tables

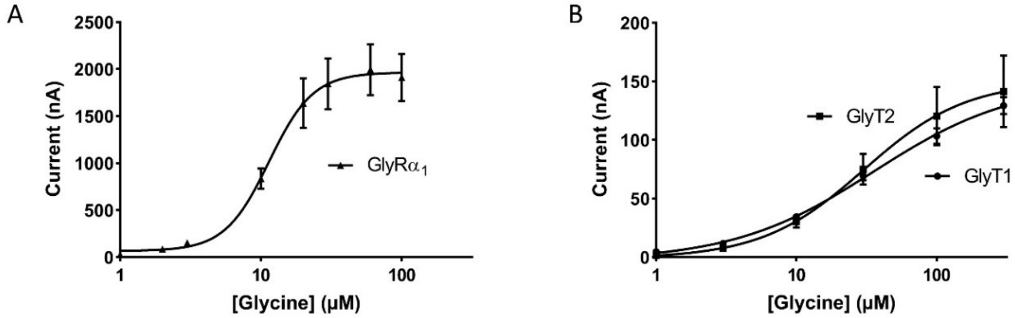

Figure 1 Currents elicited by GlyRα$_1$ are an order of magnitude greater than those elicited by GlyTs. Glycine dose-response curves for oocytes expressing only **(A)** GlyRα$_1$, **(B)** GlyT1 or GlyT2. Currents elicited by GlyRα$_1$ activation are in the thousands of nA whereas GlyT1 and GlyT2 currents are in the hundreds of nA. Raw currents were fit to the Hill equation. Symbols are mean ± SEM (*n* = 5).

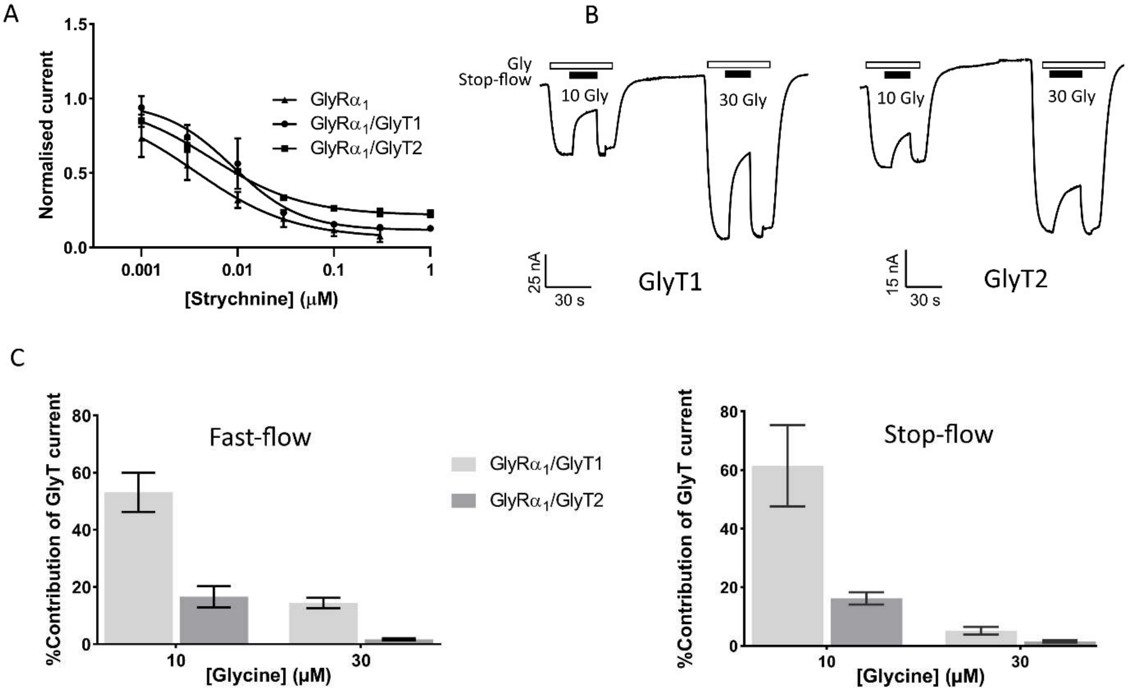

Figure 2 Contribution of GlyT current to peak current amplitude in co-expressed cells. (A) Strychnine dose-response curves for oocytes expressing GlyRα$_1$, GlyRα$_1$/GlyT1 or GlyRα$_1$/GlyT2 in the presence of 10 µM glycine. Co-expression of GlyTs with GlyRα$_1$ does not appear to affect sensitivity to strychnine. Currents were normalised to the response elicited by 10 µM glycine and fit to the Hill equation. Symbols are mean ± SEM (*n* = 3). **(B)** Example traces showing the stop-flow reduction of currents in cells expressing GlyT1 (left) and GlyT (right) alone. **(C)** The fast-flow (left) and stop-flow (right) current value recorded in the presence of strychnine was calculated as a percentage of the peak current value

without strychnine. The contribution of GlyT currents to peak currents amplitude measured in co-expressed cells decreases with increased glycine concentration. Symbols are mean ± SEM (n = 5).

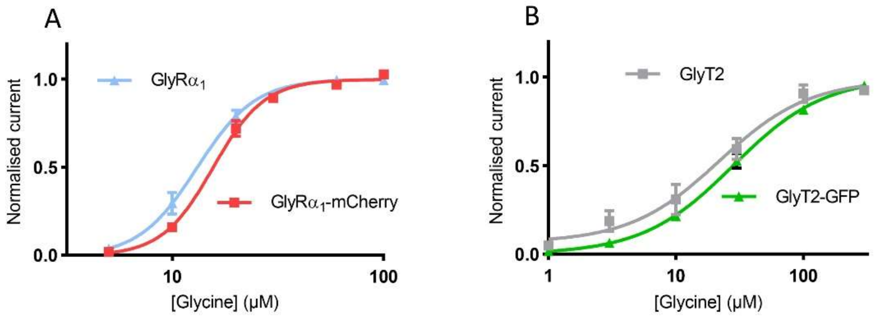

Figure 3 Fluorescent tags do not significantly change the sensitivity of GlyRα$_1$ or GlyT2 to glycine. Glycine dose responses of cells expressing **(A)** GlyRα$_1$ and GlyRα$_1$-mCherry or **(B)** GlyT2 and GlyT2-GFP show fluorescent tags do not significantly change glycine-dose response profiles. Currents were normalised to I_{max} and fit to the Hill equation. Symbols are mean ± SEM, n = 5.

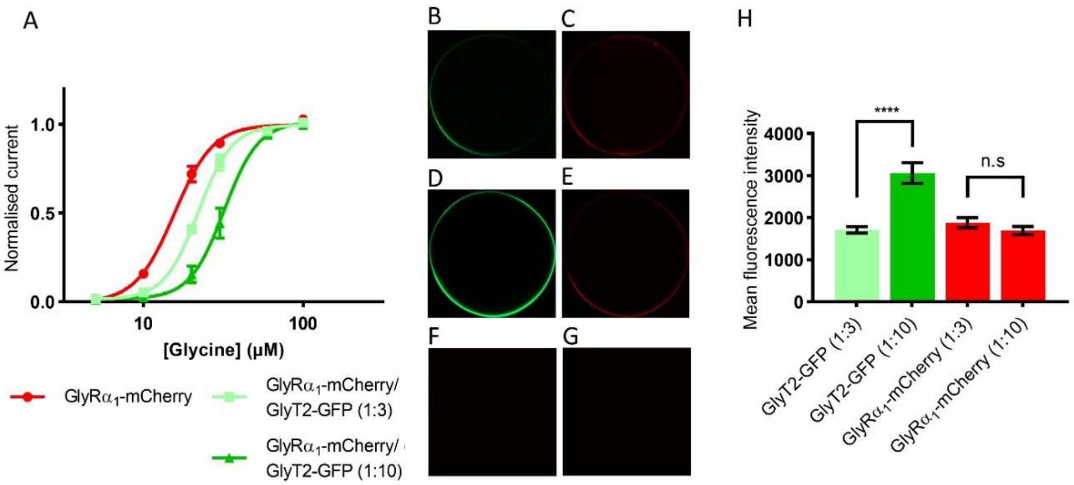

Figure 4 GlyRα$_1$-mCherry and GlyT2-GFP have similar functional properties compared to untagged proteins. GlyT2-GFP membrane surface expression increases with greater amounts of injected cRNA whereas the membrane surface expression of GlyRα$_1$-mCherry does not change. (A) Glycine dose-responses for oocytes expressing GlyRα$_1$-mCherry alone, or with different ratios of GlyT2-GFP. Currents were normalised to I_{max} and fit to the Hill equation. Symbols are mean ± SEM, n = 5. Example images of GlyT-GFP and GlyRα$_1$-mCherry expressed in the same cell for **(B, C)** 1:3 or **(D, E)** 1:10 cRNA injected ratios. **(F, G)** No fluorescence was detected from uninjected oocytes. **(H)** Mean fluorescence intensity of GlyT-GFP was significantly greater in 1:10 ratio injected oocytes compared to 1:3. There was no significant difference in GlyRα$_1$-mCherry fluorescence between 1:3 and 1:10 cRNA ratio injected oocytes. Symbols represent mean ± SEM, n = 12 **** denotes $p \leq 0.0001$ and n.s denotes $p > 0.05$.

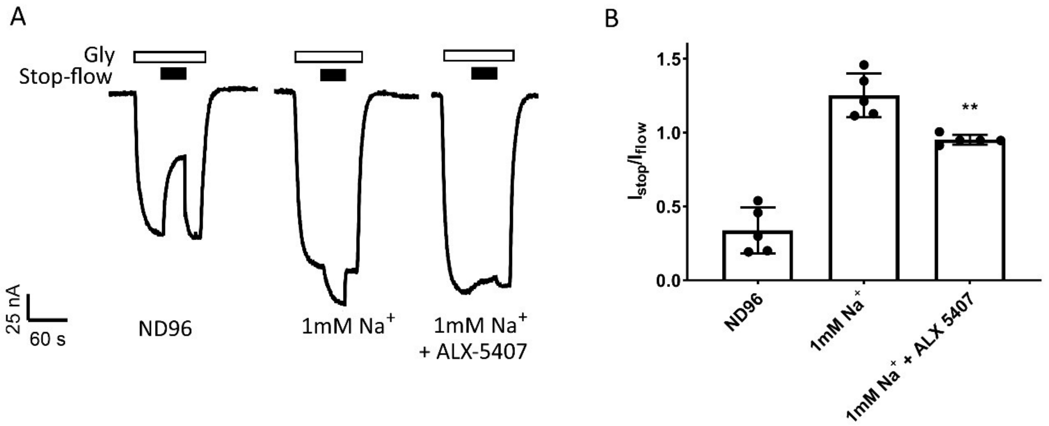

Figure 5 Glycine efflux by GlyT1 in low Na^+ can be blocked with a GlyT1 inhibitor. Application of the GlyT1 inhibitor, ALX-5407, prevents the stop-flow efflux of glycine by GlyT1 in 1mM Na^+ extracellular buffer. **(A)** Example trace showing stop flow current reduction, potentiation and no change in ND96 (96 mM Na^+) buffer (left), 1 mM Na^+ buffer (middle) and 1 mM Na^+ buffer + 1μM ALX-5407 respectively. **(B)** Histograms show normalised I_{stop}/I_{flow} values for different conditions. 1 mM Na^+ buffer + 1 μM ALX-5407 was compared to 1 mM Na^+ buffer using a one-way ANOVA and Tukey's post-hoc test. Symbols represent mean ± SEM, *n* = 5 and ** denotes $p \leq 0.01$.

Table 1 Glycine sensitivity is similar between tagged and untagged proteins

	**Glycine EC_{50} (μM)**	**95% CI**
**GlyRα_1**	13.2	12.2 – 14.3
**GlyRα_1-mCherry**	15.7	14.9 – 16.5
**GlyT2**	21.8	11.5 – 44.2
**GlyT2-GFP**	28.1	23.3 – 35.1
**GlyRα_1 / GlyT1 (1:3)**	39.5 ****	36.9 – 42.2
**GlyRα_1 / GlyT2 (1:3)**	23.0 ****	21.1 – 24.8
**GlyRα_1-mCherry/ GlyT2-GFP (1:3)**	21.9 ****	21.1 – 22.6
**GlyRα_1 / GlyT1 (1:10)**	48.8 ****	46.6 – 51.0
**GlyRα_1 / GlyT2 (1:10)**	41.6 ****	38.8 – 44.7
**GlyRα_1-mCherry/ GlyT2-GFP (1:10)**	31.3 ****	29.3 – 33.8

Glycine EC_{50} values from GlyRα_1/GlyT (1:3) or (1:10) were compared to GlyRα_1. The same comparisons were made between cells expressing corresponding fluorescently tagged proteins. Data are EC_{50} and 95% confidence interval (95% CI) ($n \geq 5$). Significance between values were tested using a one-way ANOVA and Dunnett's post-hoc test and **** denotes $p \leq 0.0001$.

Table 2 Mean fluorescence intensity of GlyRα$_1$-mCherry and GlyT2-GFP in cells expressing tagged proteins

	Mean fluorescence intensity	
	mCherry	GFP
GlyRα$_1$-mCherry/ GlyT2-GFP (1:3)	1881 ± 121	1709 ± 78
GlyRα$_1$-mCherry/ GlyT2-GFP (1:10)	1698 ± 97<br>Ns	3061 ± 244<br>****

Data is mean ± SEM (*n* = 11). Values between GlyRα$_1$-mCherry/ GlyT2-GFP (1:10) expressing cells were compared to GlyRα$_1$-mCherry/ GlyT2-GFP (1:3) expressing cells. Significance between values were tested using an unpaired t-test **** denotes $p \leq 0.0001$ and ns denotes $p > 0.05$.
